# Supplementary material for: Natural selection in the evolution of SARS-CoV-2 in bats created a generalist virus and highly capable human pathogen
Source: PLoS Biol. 2021 Mar 12;19(3):e3001115. doi: 10.1371/journal.pbio.3001115 (PMC7990310; doi:10.1371/journal.pbio.3001115)
Supplement: S1 Text — (PDF) [file pbio.3001115.s001.pdf]

S1 Text: Inferring population demography from distribution of allele frequencies.

Fitting exponential and constant population size expectations to real data

Population expansion is known to increase the relative proportion of mutations at low frequency. Following our observation that most variation in SARS-CoV-2 data sets is at very low frequency, we compared the observed distribution of SNP frequencies to expectations under two demographic models. An exponential growth model fits much better than a stable population size model, consistent with the recent spread of the virus across the globe. The fit appears to be better than that produced by Lythgoe *et al.* (2020) from within-host data. This probably reflects that within-host data is more prone to sequencing errors than consensus base calls especially at low frequencies, or perhaps reflects a super-exponential growth rate within hosts that violates the expectation given by the 1/x^2 equation (personal communication Luca Ferretti).

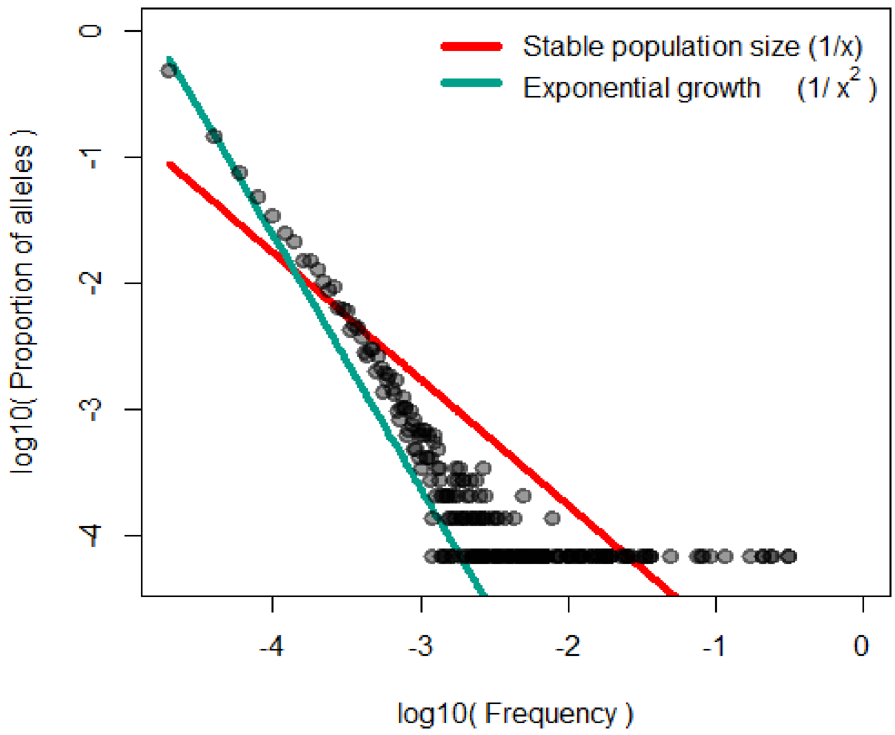

Figure A. Log-log plot of the proportion of minor alleles at each frequency, compared with expectations under two demographic models by Fay and Wu (2000) and Lythgoe *et al.* (2020). Allele frequencies were calculated across all ORFs, with each ORF separately passed through QC filters (see Methods) exact number of sequences varies across genomic regions, expectations were calculated assuming 48954 sequences, representing the median number across ORFs (data retrieved from GISAID June 28th, 2020).

References

Fay, J. C. and Wu, C. I. (2000) ‘Hitchhiking under positive Darwinian selection’, *Genetics*. Genetics Society of America, 155(3), pp. 1405–1413. Available at: https://www.ncbi.nlm.nih.gov/pmc/articles/PMC1461156/.

Lythgoe, K. A. *et al.* (2020) ‘Shared SARS-CoV-2 diversity suggests localised transmission of minority variants’, *bioRxiv*. Cold Spring Harbor Laboratory. doi: 10.1101/2020.05.28.118992.
