# Supplementary material for: Natural selection in the evolution of SARS-CoV-2 in bats created a generalist virus and highly capable human pathogen
Source: PLoS Biol. 2021 Mar 12;19(3):e3001115. doi: 10.1371/journal.pbio.3001115 (PMC7990310; doi:10.1371/journal.pbio.3001115)
Supplement: S2 Text — (PDF) [file pbio.3001115.s002.pdf]

S2 Text. Signals of positive selection in SARS-CoV-2 and frequency-based analysis of SARS-CoV-2 polymorphisms.

Signals of positive selection in SARS-CoV-2

Searching for positive selection within the SARS-CoV-2 outbreak radiation. We initially used the Bayesian FUBAR software (Murrell *et al.*, 2013) from the HyPhy package to identify sites exhibiting signatures of diversifying positive selection in the SARS-CoV-2 outbreak data. Such signatures are suggestive of the virus undergoing adaptation to humans in the pandemic. FUBAR detects positive selection by looking for codons which have an elevated rate of nonsynonymous protein coding substitutions (dN) relative to synonymous substitutions (dS). It allows both synonymous and non-synonymous rate variation across codons, as has been observed in SARS-CoV-2 evolution (Nielsen, Wang and Pipes, 2020), but assumes that selective pressures are constant through time, and across all branches (allowing no branch-to-branch variation). It estimates a posterior probability that each site is under positive selection across the phylogeny (dN/dS >1), with a posterior probability >0.9 used as the threshold for significance, as suggested by the authors (Murrell *et al.*, 2013). The input data was a concatenated coding alignment of 396 sequences from GISAID up to March 16th 2020 (S1 Table) and using a tree generated in RAxML (Stamatakis, 2014) under the GTR+Γ model. This should be a sufficient number of variants to capture the emergence of SARS-CoV-2 and any early associated adaptations. This analysis reported ten sites as showing significant evidence of positive selection across the pandemic phylogeny (Table A). Due to the low diversity in these 396 SARS-CoV-2 samples, there is limited power to confidently estimate the synonymous and nonsynonymous substitution rate for each codon. This means that statistical power to identify positive selection in the form of dN/dS>1 for any given codon is limited, and the posterior distribution should be flat. The presence of statistically significantly signatures of positive selection is therefore somewhat surprising.

Table A. The location and posterior probabilities of the ten mutations detected by the FUBAR selection analysis.

Codon in concatenated alignment	ORF	Mutation	Posterior probability
476	NSP2	I296V	0.935871674
1599	NSP3	L781F	0.943645748
3606	NSP6	L37F	0.975858139
7461	Spike	V367F	0.952007026
7708	Spike	D614G	0.939001013
7954	Spike	V860Q	0.919218684
7955	Spike	L861K	0.944083041
8720	ORF-M	D3G	0.938851732
9248	ORF-8	L84S	0.939347212
9577	ORF-N	I292T	0.952894581

As recombination is known to confound selection analyses such as for the methods in the HyPhy package (Kosakovsky Pond *et al.*, 2019), the maximum likelihood recombination detection software GARD (Kosakovsky Pond *et al.*, 2006) was used to test for recombination before performing selection analysis. This software searches for recombination by introducing potential breakpoints and optimising tree topologies either side of the new breakpoint. If the Akaike information criterion (AIC) (Akaike, 1998) is improved by the optimisations with breakpoints in, this provides significant evidence of recombination. If significant evidence of recombination is found, the method can then generate multiple non-recombinant partitions in the sequence alignment for use in downstream analyses. However, if the samples are highly related, as in the SARS-CoV-2 dataset, this phylogeny-based approach is limited in power as each recombination event introduces a large number of additional number of parameters, substantially penalising the AIC (Akaike, 1998). To detect recombination with more power for closely related samples, we also used the pairwise homoplasy index (Bruen, Philippe and Bryant, 2006), which tests for

excessive homoplasies. However, this method cannot tell if homoplasies are due to recombination or convergent evolution through parallel adaptation due to shared selection pressures.

To understand the specific mutational patterns that might explain these significant results, we looked at where in the phylogeny these putatively positively selected mutations were occurring. For all but two of the ten positive selected codons (Spike codons 860 and 861, highlighted in red in Table A), this signal was being driven by apparent convergent evolution (or homoplasy) in the tree, with the same mutation occurring in parallel across the phylogeny. To investigate whether this observation was truly due to independent events or because of recombination signatures in the SARS-CoV-2 outbreak tree, we firstly determined if the samples with these convergent mutations were geographically correlated. As selective pressure acting on an untreatable novel zoonotic virus is likely to be globally shared (adaptation to humans), but recombination requires co-localisation of viruses in the time and space, geographic clustering would be a good indication that these mutations are not independent.

The homoplasies driving ORF8 L84S and ORF1ab L1599F mutations were both found in South Korean isolates, and each of the two instances of ORF N I292T were found in the Netherlands. This geographic clustering was suggestive of recombination and was investigated further, see below.

Recombination or selection. For the two FUBAR-flagged sites, Spike codons 860 and 861, that did not show any homoplasies, both signals could be attributed to the same run of four neighbouring U to A mutations spanning the two codons. These mutations were found in only a single sample: EPI_ISL_408485 from Beijing and have not been observed since (to date 8/5/2020). This suggests that they were either sequencing errors or a large single mutation spanning two codons, which has not subsequently spread. Multiple nucleotide changes within a single codon should be rare and sequencing error is a plausible explanation.

The positive selection signature at Nsp6 codon 37 can be explained by multiple homoplasies of G to U mutations at nucleotide 11083. This mutation is found in four distinct haplotypes (Figure A) across different areas of the phylogeny. There are flanking mutations on both sides of this site shared by sequences which both possess and do not possess the 11083 mutation. For this to occur under a recombination scenario, multiple breakpoints would be required for each homoplasy. These observations therefore are not most parsimoniously explained by

recombination alone. The presence of this mutation across the tree could be driven by either positive selection, parallel sequencing error or hypermutability through polymerase slippage.

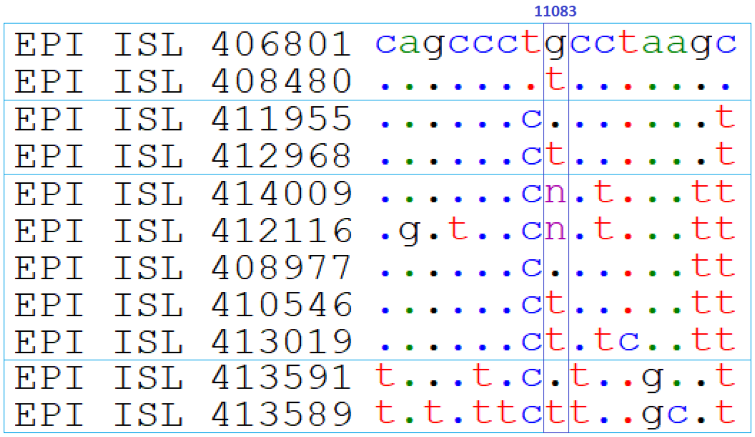

Figure A. Alignment of variable sites (removing invariant sites across these samples) surrounding the ORF1A L3606F recurrent G->T mutation. It appears to independently originate on four genetic backgrounds. The presence of undetermined nucleotides, ‘n’s, in a few of the sequences suggests that sequencing ambiguity is common in this region.

Both the ORF8 codon 84 and ORF1ab codon 1599 positive selection signals appear to be due to a single South Korean sample (GISAID accession 413017). This sample possesses two derived mutations either side of a hypothesised breakpoint. These pairs of derived mutations belong to samples with different haplotypes (Figure B). Therefore the 413017 sample appears to be a recombinant between sample 413018 and 413513 or 412871. As both 413017 and 413018 were sequenced by the same laboratory and released at the same time, this recombination event may be an artefactual product of laboratory cross-contamination.

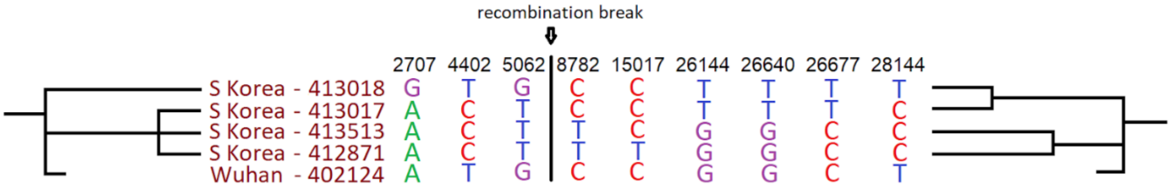

Figure B. Alignment of variable sites in the whole genome alignment, with base positions shown above. The putative recombinant South Korean sample 413017 shows mismatching topologies across its genome, clustering with different South Korean samples either side of the inferred breakpoint. The Wuhan sample 402124 was collected on 30/12/2019, and shows no unique mutations, it should thus represent the ancestral state of the four chosen samples.

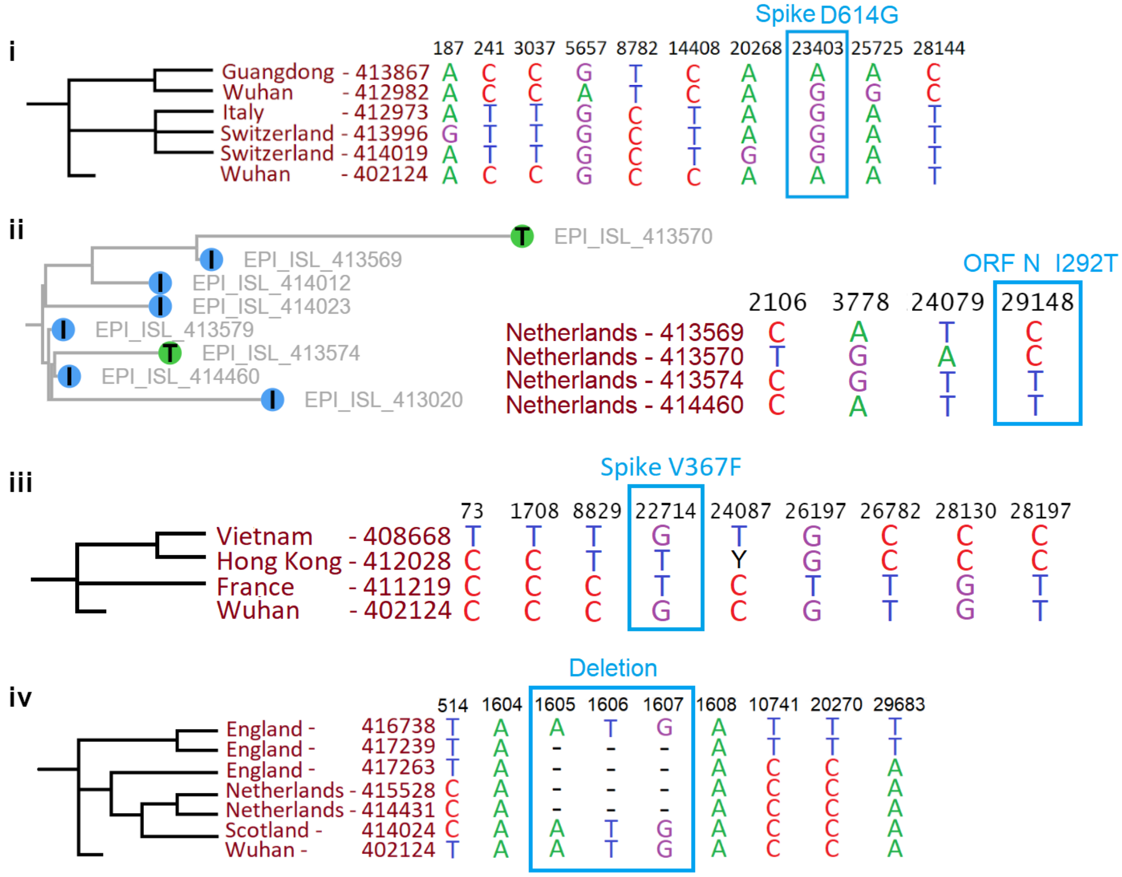

Figure C. (i) Spike D614G replacement: sites either side of D614G show derived mutations in Wuhan 412982 congruent with the tree, suggesting that it is not a recombinant, or has multiple breakpoints. **(ii)** N ORF I292T: both Netherlands samples with the mutation were sequenced by the same Dutch laboratory and released at the same time. **(iii)** Spike V367F homoplasy at a single site in Hong Kong, unlikely to be a recombinant. **(iv)** An observed insertion homoplasy in newer data.

The Spike D614G signal was driven by apparent convergent evolution between one Wuhan sample (412982) in addition to the main lineage containing 86 samples. This sample shares mutations with its closest related sequence (Guangdong 413867) on both sides of this homoplasy (Figure C(i)), suggesting it is not the result of recombination. No newly sequenced samples uploaded up to 27/4/2020 containing the D614G mutation clustered with the Wuhan 412982 sample, suggesting that this haplotype did not spread or that this homoplasy is driven by sequencing error. Additional sequences displaying apparent convergent evolution at this site have since been sequenced, these have been taken as evidence of positive selection (Phelan *et al.*, 2020). However, given that this mutation now occurs in 59% of sequenced samples (as of the

27th of April 2020), it will be one of the mutations most likely to be variable if multiple viral genotypes are present following laboratory contamination or in mixed infections, and so most prone to being shuttled onto new backgrounds by recombination. Therefore, whilst high frequency mutations are the most important to study, they are also the most prone to misleading homoplasies, and must be analysed with the most caution.

The N ORF 292 site detected by FUBAR is driven by a similar convergent evolution event history. However, both samples exhibiting the same derived I to T mutation (Figure C(ii); GISAID IDs 413570 and 413574) were sequenced by the same Dutch laboratory and released at the same time, again suggesting that laboratory cross contamination is a likely driver. However, unlike South Korean sample 413017, there is only one shared derived mutation (codon 292), and therefore the genomic evidence for recombination in these samples is weaker.

The ORF M D3G mutation was found in three samples, 414010, 414017, and 413999, from England, Scotland, and Switzerland, respectively. The positive selection signal was driven by sample 414010 from England, which exhibited a homoplasy at ORF N codon 156, representing the A156S mutation, which is shared with two samples from the Netherlands 414450 and 414457. More recent samples since this analysis, which exhibit both the ORF M D3G and ORF N A156S mutation, have been sequenced in England (e.g., 449635) suggesting that this sample represents a true recombination/convergent evolution event which was transmitted. Which of these two codons represents the convergent mutation/recombination event is unclear due to the low levels of divergence between lineages at the early stage of the pandemic when this event occurred. Additionally, samples exhibiting only one of ORF M D3G or N A156S, but not both, are observed in more recent sequencing data, e.g., English sample 461979 for the former and Indian sample 475029 for the latter.

The Spike V367F replacement signal was driven by apparent convergent evolution between four French samples sequenced in January and a Hong Kong sample 412028, which shows shared variation either side of the homoplasy suggesting it is not a recombinant (Figure C(iii)). Looking through more recent data shows additional homoplasies in a neighbour joining tree. Additionally, newly generated sequences since the FUBAR analysis cluster around the Hong Kong sample, further suggesting it is not a laboratory generated sequencing error. This site was also flagged in our updated methodology in mid-May (main text Fig 2E), however this substitution has not been seen for months, suggesting that it stochastically been lost. It might be speculated that the multiple

origins of this amino acid replacement might be driven by positive selection within hosts, and its loss may have been driven by negative selection between hosts. Trade-offs of this kind have been observed in HIV-1 (Theys *et al.*, 2018).

Subsequent scans of newer data have revealed additional evidence of laboratory recombination events (Figure C). Given these observed issues with the data, it is clear that analysis for positive selection should not consider terminal branches in searching for dN/dS>1, as these are prone to sequencing error artefacts, and analyses should instead only utilise internal branches.

Frequency-based analysis of SARS-CoV-2 polymorphisms

In addition to searching for positive selection, we investigated if signatures of purifying selection on segregating variation in the current SARS-CoV-2 data could be observed (sequences as of 14/5/20). We compared the relative frequencies of nonsynonymous and synonymous mutations in the pandemic data. Codons with multiple mutations present were discarded from the analysis to avoid ambiguity in the order of mutations and simplify synonymous/nonsynonymous classification.

Most mutations of both classes are at very low frequency (main text Fig 2), indicative of the viral population expansion that the pandemic has undergone. dN/dS was approximately 0.6 in singletons, suggesting that 40% of nonsynonymous mutations are strongly deleterious and therefore never observed in the population. There is a weak observable trend towards a higher proportion of mutations being synonymous at the highest frequency intervals, suggestive of some ongoing selection against circulating amino acid replacements in the pandemic. This observation may be partially driven by sequencing errors which are not transmitted and so are at low frequency. These sequencing errors are likely to have a dN/dS value of 1, which may make the estimate that 40% amino acid replacements are strongly deleterious an underestimate of the true value. However, the decline in nonsynonymous/synonymous ratio occurs across the range of frequencies, suggesting that sequencing errors alone are not driving the trend. It is important to consider that the observed frequencies are likely to differ from true global frequencies due to biased sampling of infections in the pandemic (Maclean *et al.*, 2020), and so we caution against overinterpretation of specific mutation frequencies.

References

Akaike, H. (1998) 'Information Theory and an Extension of the Maximum Likelihood Principle', in *Selected Papers of Hirotugu Akaike*. Springer, New York, NY, pp. 199–213. doi: 10.1007/978-1-4612-1694-0_15.

Bruen, T. C., Philippe, H. and Bryant, D. (2006) 'A simple and robust statistical test for detecting the presence of recombination', *Genetics*. 172(4), pp. 2665–2681. doi: 10.1534/genetics.105.048975.

Kosakovsky Pond, S. L. *et al.* (2019) 'HyPhy 2.5-A Customizable Platform for Evolutionary Hypothesis Testing Using Phylogenies', *Molecular Biology and Evolution*, 37(1), pp. 295–299. doi: 10.1093/molbev/msz197.

Kosakovsky, S. L. *et al.* (2006) 'GARD: a genetic algorithm for recombination detection', *Bioinformatics Applications Note*, 22(24), pp. 3096–3098. doi: 10.1093/bioinformatics/btl474.

Maclean, O. A. *et al.* (2020) 'No evidence for distinct types in the evolution of SARS-CoV-2', *Virus Evolution*, 6(1), p. veaa034. doi: 10.1093/ve/veaa034.

Murrell, B. *et al.* (2013) 'FUBAR: A fast, unconstrained bayesian AppRoximation for inferring selection', *Molecular Biology and Evolution*. doi: 10.1093/molbev/mst030.

Nielsen, R., Wang, H. and Pipes, L. (2020) 'Synonymous mutations and the molecular evolution of SARS-Cov-2 origins', *bioRxiv*. Cold Spring Harbor Laboratory. doi: 10.1101/2020.04.20.052019.

Phelan, J. *et al.* (2020) 'Controlling the SARS-CoV-2 outbreak, insights from large scale whole genome sequences generated across the world', *bioRxiv*. Cold Spring Harbor Laboratory, p. 2020.04.28.066977. doi: 10.1101/2020.04.28.066977.

Stamatakis, A. (2014) 'RAxML version 8: a tool for phylogenetic analysis and post-analysis of large phylogenies', *Bioinformatics Applications*, 30(9), pp. 1312–1313. doi: 10.1093/bioinformatics/btu033.

Theys, K. *et al.* (2018) 'The impact of HIV-1 within-host evolution on transmission dynamics', *Current Opinion in Virology*. Elsevier B.V., pp. 92–101. doi: 10.1016/j.coviro.2017.12.001.
