# Supplementary material for: Natural selection in the evolution of SARS-CoV-2 in bats created a generalist virus and highly capable human pathogen
Source: PLoS Biol. 2021 Mar 12;19(3):e3001115. doi: 10.1371/journal.pbio.3001115 (PMC7990310; doi:10.1371/journal.pbio.3001115)
Supplement: S3 Text — (PDF) [file pbio.3001115.s003.pdf]

S3 Text. Model selection when testing for a nCoV-specific rate change.

In our Bayesian phylogenetic analysis of the nCoV clade we focus on applying a local clock model to the branch leading to the nCoV clade. We had not performed a full model comparison of available clock models because our aim is to test a specific prior hypothesis motivated by the CpG adaptive shift we identify and the selection analyses. It is likely that an uncorrelated relaxed clock would fit the data best because of its flexibility in modelling rate variation among all branches. However, it does not serve our purposes as it does not allow testing for an elevated rate on a specific branch or set of branches. In addition, the fact that rates are governed by a specific distribution (e.g., an exponential or lognormal distribution) results in a smoothing effect of discrete rate variation (Worobey, Han and Rambaut, 2014). In Figure A we summarize such an analysis for the NRR2 alignment and compare it to a non-clock maximum likelihood tree that clearly shows the higher root-to-tip divergence for the nCoV taxa.

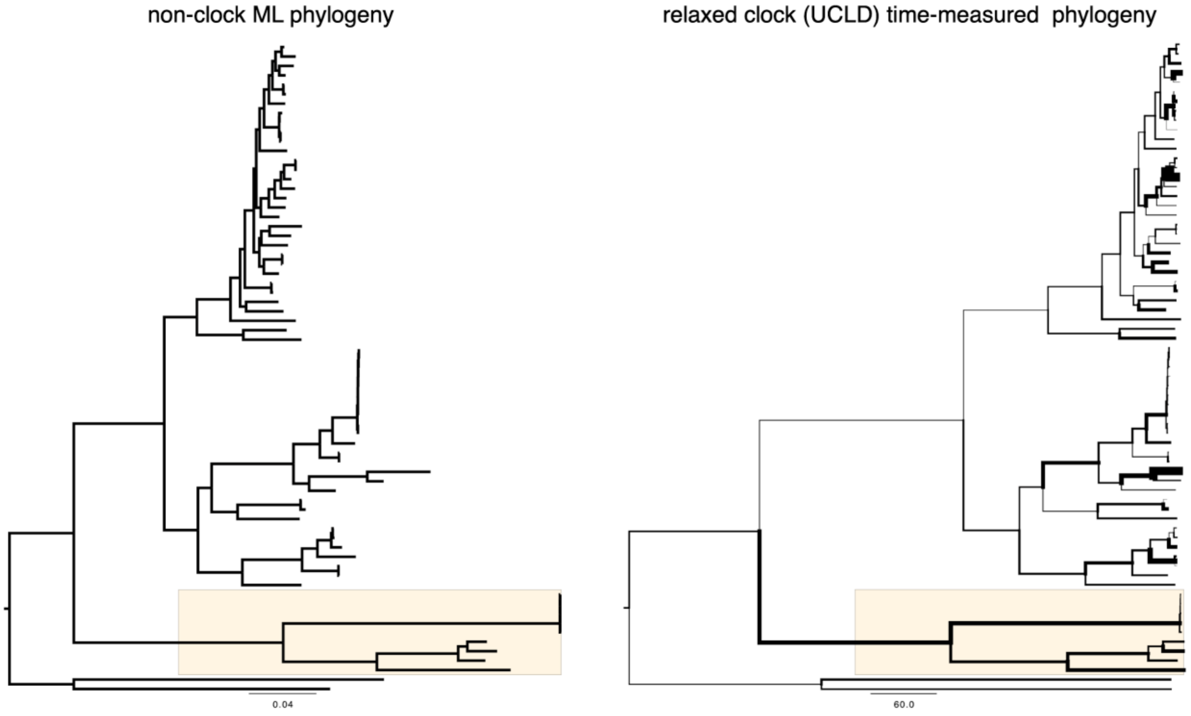

Figure A. NNR2 non-clock maximum likelihood phylogeny (left) and time-measured BEAST MCC tree (right), summarized from an uncorrelated lognormal distribution (UCLD) relaxed clock model. The nCoV clade is highlighted by a yellow box. The rate variation among branches in the UCLD MCC tree is represented by the branch thickness.

The extended root-to-tip divergences for the nCoV taxa also require an elevated rate on the ancestral branch of the clade. In fact, this branch accommodates one of the fastest rates on internal branches and it stands out as a relatively long internal branch with a high rate. Some other branches in the nCoV clade also appear to be associated with a high rate under the UCLD relaxed clock model. This could be related to the smoothing effect we mention above. We also fitted a clade-wide local clock model and attempted to compare model fit using marginal likelihoods, but we could not identify a meaningful difference in model fit. This is not surprising

because the contemporaneous data cannot distinguish between a strongly elevated rate on the shared nCoV ancestral branch or an overall elevated rate over longer time (the entire nCoV clade). For this reason, we now also include the results of a clade-specific local clock in Table A. The estimates under a clade-specific local clock also suggest a higher rate for the entire nCoV rate but the difference is less pronounced compared to the branch-specific local clock as expected. Because the branch-specific local clock model fits the prior hypothesis we aim to test, we opted to present the results of this model (for NRR2) in Fig 3A (main text). The NRR1 and NRR2 phylogenies for the remaining models are presented in Figure B.

Table A. Local clock model and mean divergence time estimates with 95% highest posterior density intervals for key nodes in the nCoV lineage for NRR1 and NNR2.

**Alignment**	**NRR1**		**NNR2**	
**Local model**	nCoV branch	nCoV clade	nCoV branch	nCoV clade
**Background rate (95% HPD)**	0.000238 (0.000146,0.000386)	0.000229 (0.00137,0.000314)	0.000281 (0.000185,0.000372)	0.000266 (0.000170,0.000356)
**nCoV rate (95% HPD)**	0.000722 (0.000386,0.001089)	0.000368 (0.000227,0.000516)	0.00283 (0.001022,0.004825)	0.000539 (0.000343,0.000734)
**TMRCA(nCoV)**	1189 (254,1538)	1480 (1675,1237)	1467 (1264,1641)	1713 (1820,1593)
**TMRCA(Guangdong pangolin/SC2)**	1664 (1280,1815)	1789 (1872,1685)	1732 (1627,1825)	1866 (1915,1801)
**TMRCA(RaTG13/SC2)**	1924 (1810,1958)	1956 (1979,1929)	1955 (1932,1976)	1984 (1996,1970)
**TMRCA(RmYN02/SC2)**	1949 (1916,1975)	1973 (1990,1952)	1976 (1959,1991)	1996 (2004,1986)

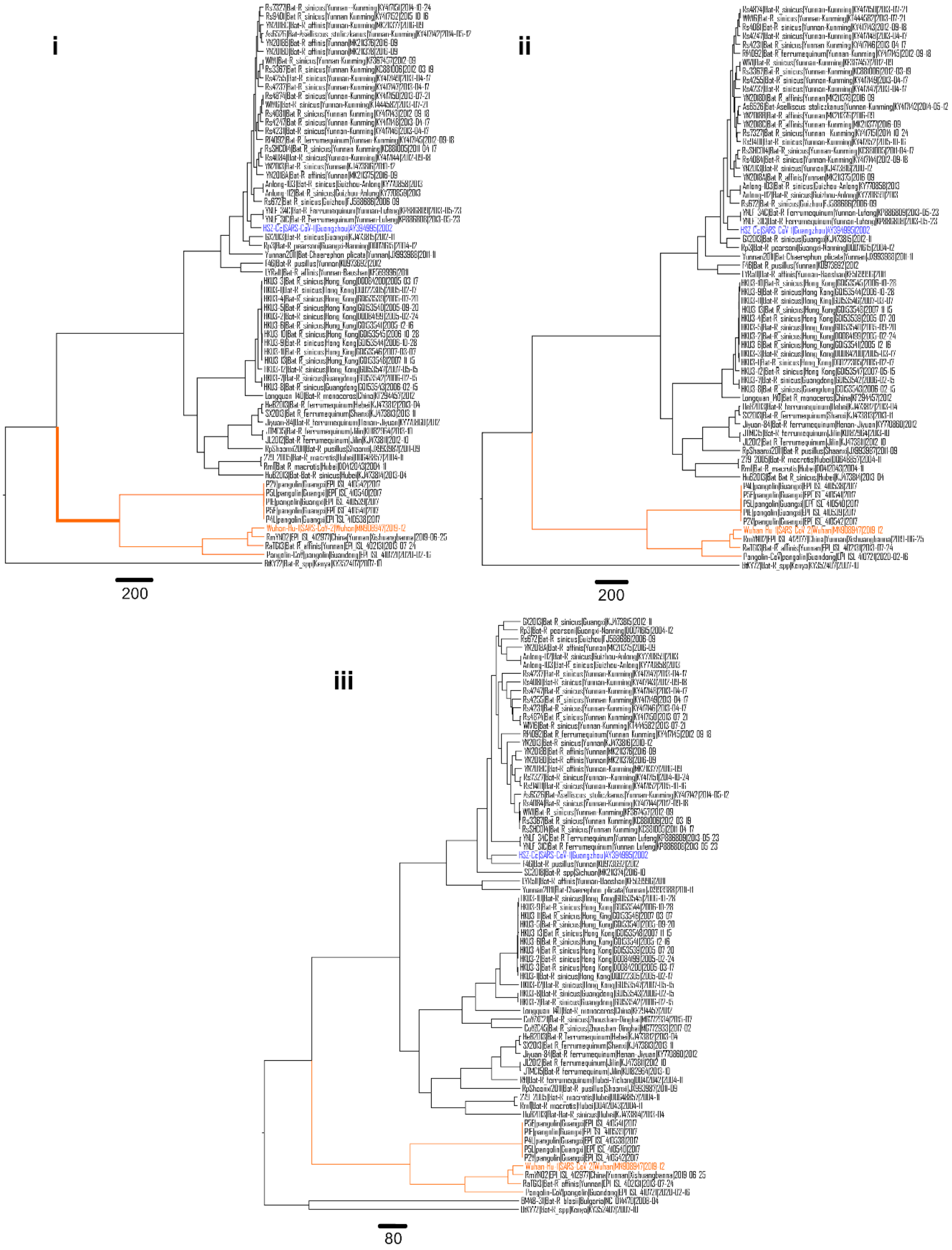

Figure B. BEAST phylogenies with a separate local molecular clock model applied on the nCoV clade base branch (bold) using (**i**) NRR1, and with the separate clock model applied on all branches of the nCoV clade using (**ii**) NRR1 and (**iii**) NRR2. The nCoV clade and SARS-CoV-2 are coloured in orange, SARS-CoV-1 is coloured in blue.

References

Worobey, M., Han, G. Z. and Rambaut, A. (2014) ‘A synchronized global sweep of the internal genes of modern avian influenza virus’, *Nature*., 508(7495), pp. 254–257. doi: 10.1038/nature13016.
