# Supplementary figures and images for: Natural selection in the evolution of SARS-CoV-2 in bats created a generalist virus and highly capable human pathogen
Source: PLoS Biol. 2021 Mar 12;19(3):e3001115. doi: 10.1371/journal.pbio.3001115 (PMC7990310; doi:10.1371/journal.pbio.3001115)

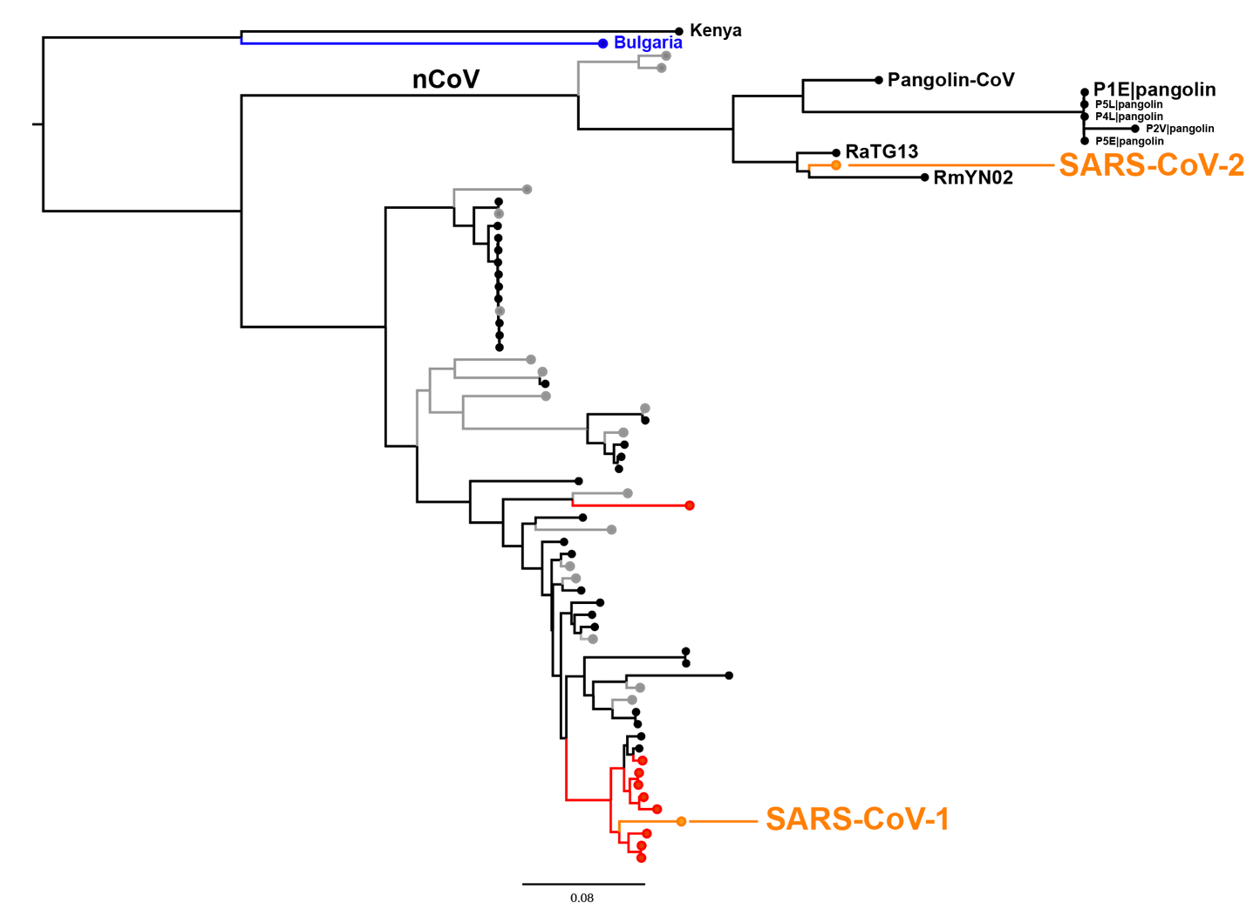

Supplement: S1 Fig — Grey, red, and blue variants are coloured according to Letko and colleagues [12] who showed experimentally some viruses are able to use human ACE2 (red), while some require exogenous protease treatment in vitro (grey); the red outlier is a known recombinant. Black indicates not tested by Letko and colleagues, while the virus in blue (sampled in Bulgaria) could not be induced to infect human cells. The scale bar corresponds to nucleotide substitutions per site. (PNG) [file pbio.3001115.s004.png]

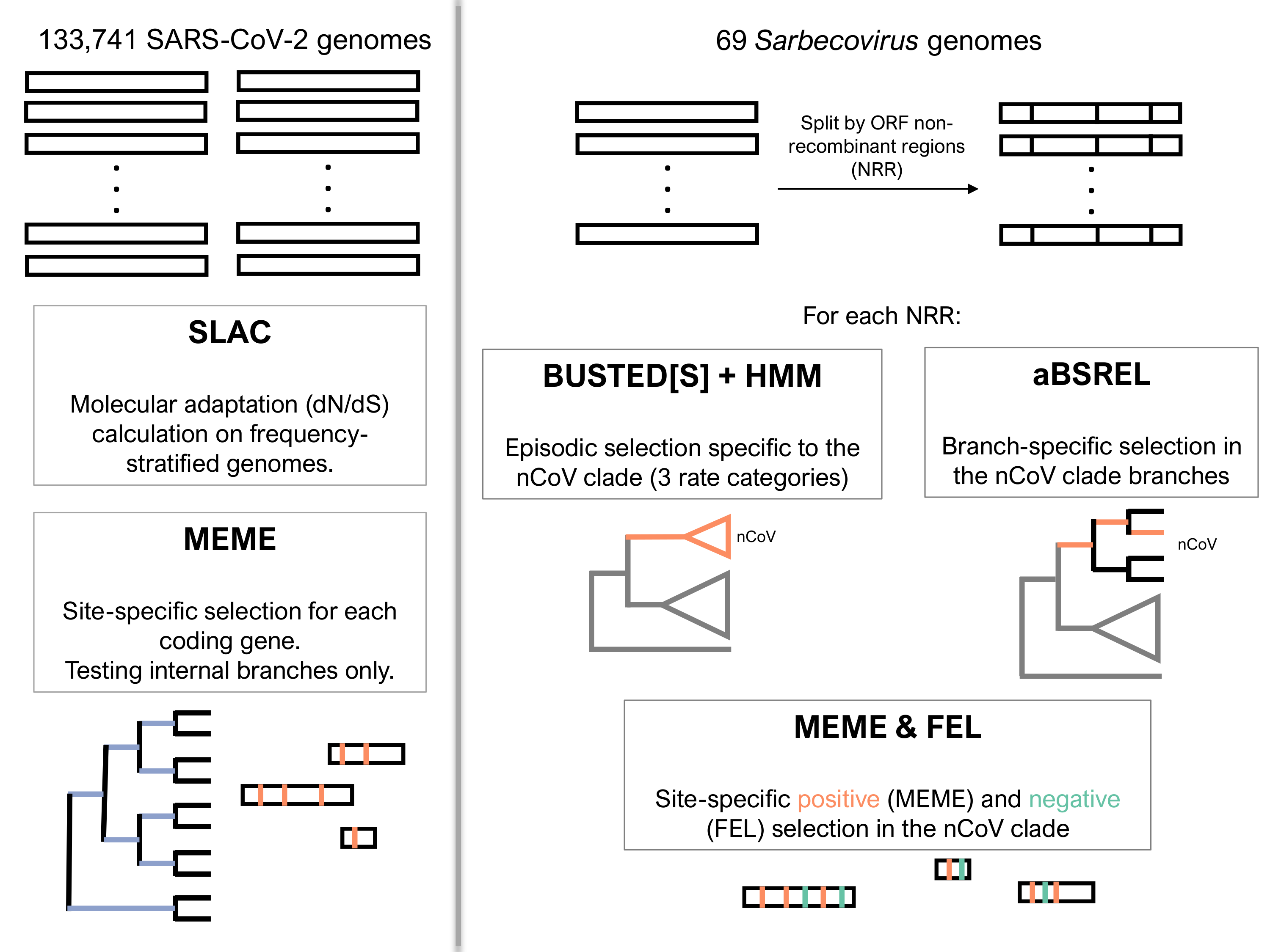

Supplement: S2 Fig — Schematic representation of selection methods implemented on each data set of the analysis: (i) the 133,741 SARS-CoV-2 genomes and (ii) the 69 Sarbecovirus genomes. (PNG) [file pbio.3001115.s005.png]

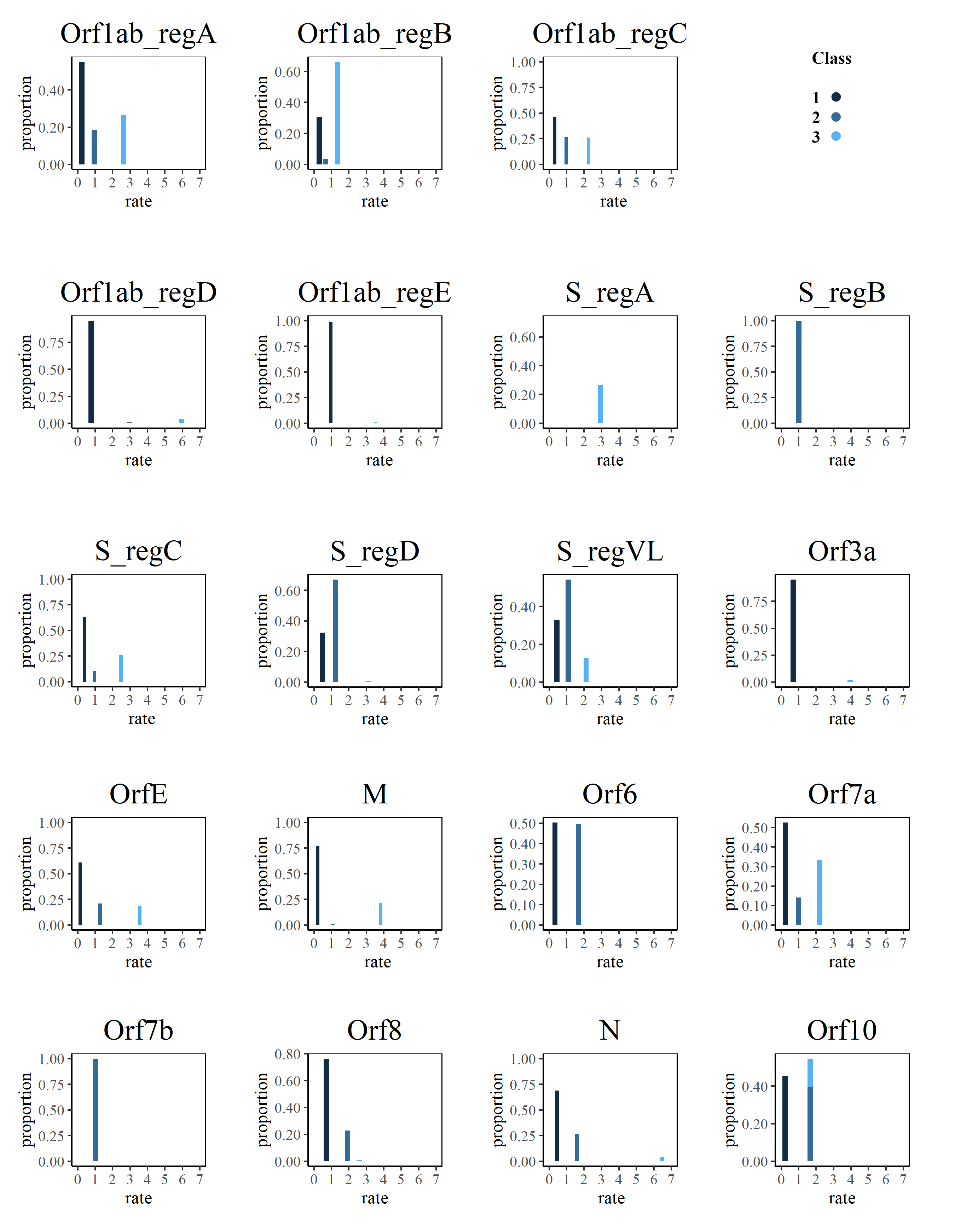

Supplement: S3 Fig — (PNG) [file pbio.3001115.s006.png]

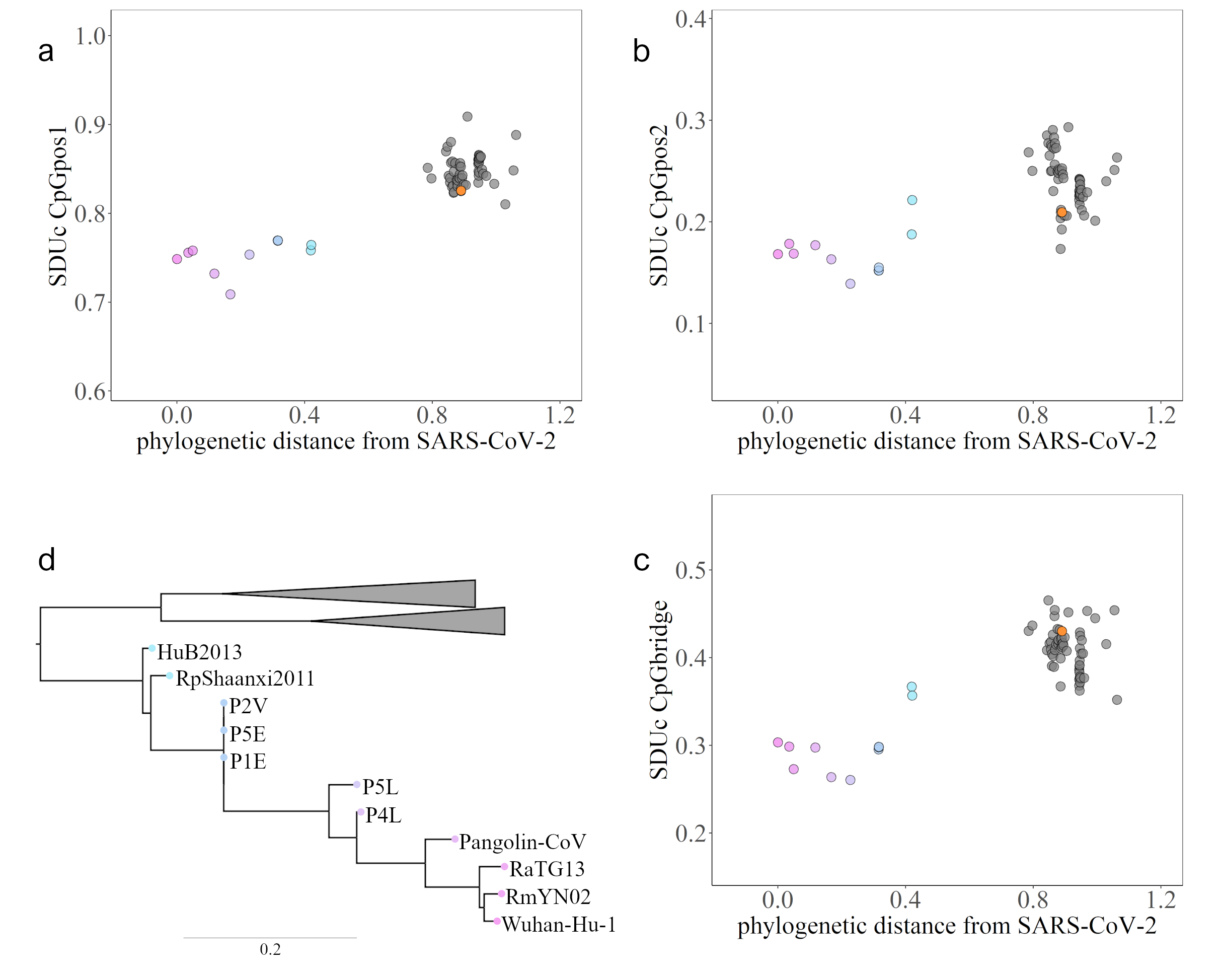

Supplement: S4 Fig — Corrected synonymous dinucleotide usage (SDUc) values for the Orf1ab of each Sarbecovirus for all dinucleotide frame positions: (A) pos1: first and second codon positions, (B) pos2: second and third codon positions, and (C) bridge: third codon position and first position of the next codon, plotted against patristic distance from SARS-CoV-2 (reference genome Wuhan-Hu-1). (D) The tip colours of the phylogeny correspond to the SDUc data points. SARS-CoV-1 is labelled in orange in panels A, B, and C for comparison. The non-nCoV part of the phylogeny has been collapsed for clarity. (PNG) [file pbio.3001115.s007.png]

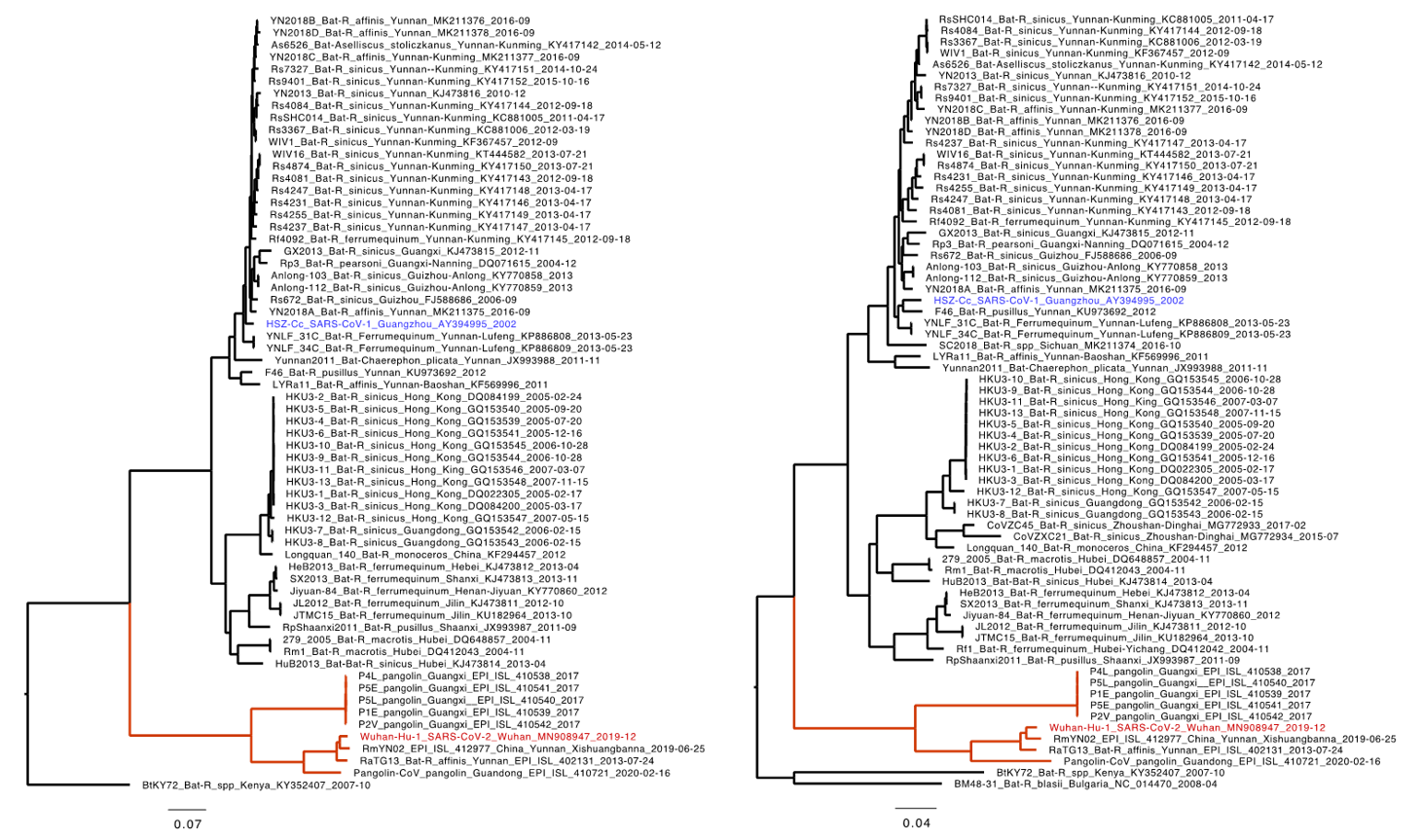

Supplement: S5 Fig — The trees were inferred using IQTREE using a GTR substitution model with gamma-distributed rate variation among sites. The nCoV lineage is indicated in red. SARS and SARS-CoV-2 are shaded in blue and red, respectively. (PNG) [file pbio.3001115.s008.png]

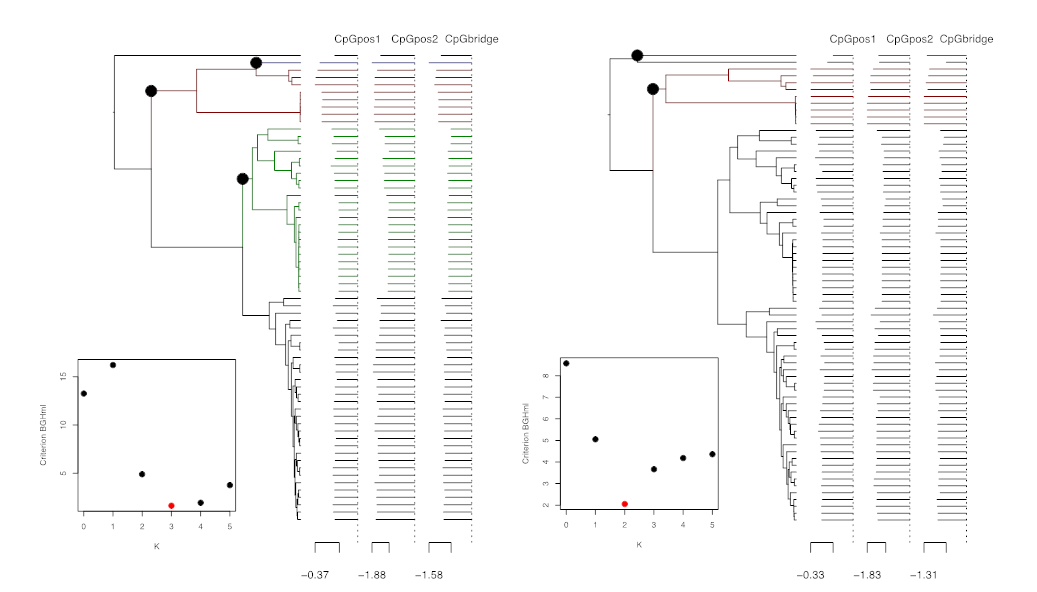

Supplement: S6 Fig — The log-transformed CpG content values are shown at the tips of the trees. The identified shifts are indicated with black circles on their respective branches and with different colours for the lineages and CpG measures involved. The inset shows the results for penalized least-squares model selection criterion (BGHml). (PNG) [file pbio.3001115.s009.png]
