# Supplementary material for: Natural selection in the evolution of SARS-CoV-2 in bats created a generalist virus and highly capable human pathogen
Source: PLoS Biol. 2021 Mar 12;19(3):e3001115. doi: 10.1371/journal.pbio.3001115 (PMC7990310; doi:10.1371/journal.pbio.3001115)
Supplement: Attachment — Submitted filename: MacLean-Lytras-etal-reviewers-response-24thDec.pdf [file pbio.3001115.s017.pdf]

Response to reviewers: PBIOLOGY-D-20-03122R1

Natural selection in the evolution of SARS-CoV-2 in bats created a generalist virus and highly capable human pathogen

Oscar A. MacLean, Spyros Lytras, Steven Weaver, Joshua B. Singer, Maciej F. Boni, Philippe Lemey, Sergei L. Kosakovsky Pond, David L. Robertson

Reviewer #1:

This manuscript by MacLean/Lytras and colleagues is a fascinating analysis assessing the nature of selection occurring in horseshoe bats compared to SARS-CoV-2 evolution in humans. In this study the authors explore the evolutionary history of bat Sarbecoviruses that may help shed some insight into the emergence and rapid spread of SARS-CoV-2. One of the quirks of this pandemic is that the virus responsible evolves relatively slowly resulting in a very shallow phylogenetic tree. This feature inhibits the statistical power of standard comparative methods and as such other computational approaches are needed. This manuscript adapts a rigorous methodology to measure the strength and direction of selection from SARS-CoV-2 and its Bat precursors while carefully considering confounding errors (sequencing or other lab-based errors) that may have occurred on terminal branches.

One thing that does irk me slightly is the authors assertions of the "generalist" nature of these viruses allowing for efficient spillover events. While there is no doubt that SARS-CoV-2 is well adapted for humans and the authors have not found any evidence for selection in the ancestral branches leading to SARS-CoV-2 there is a huge amount of under sampling in wildlife species so a greater sampling of animal species in nature including bats could indicate a closer ancestor and may well demonstrate selection and host-adaptations. This caveat and the paucity of SARS-CoV-2 genomes from very early in the pandemic from China should be duly recognized within the text. Moreover, given the divergence estimates from RmYN02/SARS-CoV-2 of 1976 there is potentially decades of unobserved evolution that may have occurred that warrants consideration.

We want to thank the reviewer for their positive and constructive feedback. We believe that we have now addressed all comments to the best of our ability and the changes have very much improved the quality of the manuscript. In response to the under-sampling and lack of very early SARS-CoV-2 genomes caveats requested, we have add some text to our discussion on this point and how these might affect our analysis and interpretation.

Specific comments.

1. The methods are quite technical and for a broader reader of PLoS Biology I think they would find it challenging to understand the nuances of each computational approach. It may be worth the authors considering a figure (even as supplementary) to highlight the methodological approach used.

We appreciate the need to clarify some of the more technical parts of our methodology. We now include a supplementary figure (S2 Fig) that should hopefully provide a straightforward and easy to interpret visual description of our selection methodology to the broader readers of the journal. We would be happy to include this in the main text if that would be preferred.

2. The authors premise that the majority of host adaptations occurred before the emergence of SARS-CoV-2 in humans while may be technically sound with the current sequence data

but how can the authors dismiss the hypothesis that human-specific adaptation would have likely reached fixation even before the first SARS-CoV-2 genome was sequenced?

The under-sampling of related *Sarbecoviruses* and inherent lack of sequences from the first SARS-CoV-2 genomes that emerged in humans are inevitable problems for our analysis. There's a good chance this issue will never be solved as some time has passed since the initial spill-over to humans. Nonetheless, as we now further explain in our discussion, our *Sarbecovirus* branch-specific selection analysis does not pick up any signal of positive selection at the terminal branch leading to SARS-CoV-2 for any of the non-recombinant ORF regions. Even though the analysis is limited by the lack of closer virus relatives – if substantial adaptive change were to have taken place right before or right after SARS-CoV-2 emerged in humans – there should be signal in the terminal branches, and according to the currently available data that is not the case. Our updated discussion now has the caveat that the possibility of early changes in humans cannot be 100% dismissed, in particular:

"The amount of time between the initial transmission of the virus to humans and sequencing the first SARS-CoV-2 remains unknown. ... indicating that substantial adaptation to humans is unlikely to be required for these nCoV viruses to cause a pandemic."

That SARS-CoV-2 can readily transmit to other animals (minks, cats etc.) is strongly indicative of this point as it is very unlikely the generalist property evolved as a consequence of adaptation to human-human transmission.

3. As this field is continuously evolving with new genomic data being added daily the statement in line 328-331 should no longer be considered accurate as we can now observe rapid adaptation of SARS-CoV-2 in mink populations. E.g Y453F in the receptor biding motif and this rarely occurs in humans. My point linked with the above point is that increased surveillance permitted us to observe this variant in minks while similarly changes may have occurred earlier in the pandemic and become fixed before sequencing was done. Could rapid adaptation in late 2019 from unsampled asymptomatic transmission chains be plausible?

We agree with the reviewer that the previous phrasing of this sentence did not successfully convey the message we wanted to communicate and we have now modified it. Still, even if there is evidence of early adaptation after cross-species transmission, for example in minks, there are very few changes involved and mainly related to optimisation of ACE2 binding rather than 'gain-of-function'. Furthermore, these early changes do not alter the fact that these viruses were able to transmit to these species and spread; and be transmitted back to humans. The most common mink-associated change, S:Y453F, has been seen in nearly 1000 SARS-CoV-2s in humans. We have added a new discussion paragraph following the relevant part of the section where we discuss the important point the reviewer raises here and how our analysis and results link to it.

4. Unsurprisingly, the authors find that up until early June, relatively weak purifying selection was acting on SARS-CoV-2 sequences. If the authors extended their analysis to the present what would they expect to find given that there is more diversity in current circulating variants?

We have expanded the analysis to include sequences up to mid-October. The overall patterns are largely unchanged. There are some indications of increased selective pressure

in later samples, but that is also expected to occur once the viral population begins to experience more immunological and other selective pressures and the reviewer is absolutely correct on this point. The arising now of more mutated variants, for example, the UK lineage B.1.1.7 appear to be associated with evolution in the context of chronic infections (discussed here https://virological.org/t/preliminary-genomic-characterisation-of-an-emergent-sars-cov-2-lineage-in-the-uk-defined-by-a-novel-set-of-spike-mutations/563) but as this encompasses a different evolutionary process (chronic versus acute-infection associated evolution) we feel it is beyond the scope of this manuscript to introduce it, as we're focusing on the first 11 months of the pandemic. If the reviewer feels strongly about this, we would be more than happy to add a new section to our paper.

5. While SARS-CoV-2 consensus sequences remain largely unchanged over time within hosts is there evidence for a more dynamic evolutionary process sub-consensus?

The tendency for transmitted sequences to change little from the 'consensus' arises because SARS-CoV-2 is principally an acute virus, even in the vast majority of severe cases. Coupled with the ability to correct replication errors this leads to a very low rate of evolution. There is some evidence that SARS-CoV-2 develops intra-host variation during infection (e.g., https://www.biorxiv.org/content/10.1101/2020.05.28.118992v4) but these studies confirm low levels of diversity and strong constraints as we find at the population scale. Chronic SARS-CoV-2 evolution, in particular in immunocompromised individuals, appears to be different with more dynamic evolutionary processes playing out. We feel, however, the sparse nature of such infections, let alone available datasets put this beyond the scope of our paper.

6. The authors use MEME as documented in Figure 1c to individual sites subjected to episodic diversifying selection. However, I thought that MEME assumes that selective pressures between branches are uncorrelated. Surely, this is not the case for SARS-CoV-2 as changes are occurring very slowly across the phylogeny as neighboring branches will be correlated. Have the authors considered a mixed effects covarion model and if this would improve power to detect directional selection?

This is an intriguing suggestion, but we do not believe that it would lead to dramatically improved power and removing the independence assumption will render the current computational (efficient) framework inapplicable. Firstly, to our knowledge, a fixed site effects type model in the covarion framework has not been published: it has generally been used on whole alignments as a random-effect model, and site-level inference is done post-hoc (e.g., using an empirical Bayes type approach). Secondly, in a direct comparison of the covarion and unrestricted branch site models (like those used in MEME) on gene-level analyses, we found that covarion type models did not show greater power (they had lower power), but were prone to above-nominal false positives in certain settings (Figure 2 in https://doi.org/10.1093/molbev/msv035). Part of this can be attributed to the fact that a part of the state space for the covarion models (evolutionary "mode") is never observable, even at the leaves of the tree. Thirdly, it is difficult to make any prediction about how selective regimes in a virus would be correlated: what the reviewer suggests is plausible, but so is an alternative model where jumping from host to host exposes the virus to different (and uncorrelated) selective environments viz-a-viz immune responses.

7. The addition of synonymous site rate variation is a great addition to BUSTED as constant dS rates can elevate false-positive and reduce power to test individual sites for selection.

However, can the authors comment on whether accommodating synonymous rate variation results in reduced power compared to the original method?

When the extent of synonymous rate variation (SRV) is low to moderate, power loss is minimal (see Figure 4 in https://doi.org/10.1093/molbev/msaa037). However, not including SRV in the model leads to an "*uncontrolled rate of false positives*" even for moderate levels of SRV. Considering that the estimated extent of SRV in SARS-CoV-2 data is not small, including SRV is essential.

8. How did the authors consider multinucleotide mutations in their analysis as I am sure the authors are aware that there is a high possibility of false positives with branch sites tests like BUSTED. For example, Venkat et al. (2018).

This is indeed a concern; the Venkat et al (2018) paper is rather narrowly focused on single branch tests that are short (e.g., the human lineage in Nielsen-Yang style branch site models), but we and others agree than MNM needs to be better accounted for (e.g., https://www.biorxiv.org/content/10.1101/2020.05.13.091652v1.full.pdf). We previously implemented a version of BUSTED that allows for MNM (https://github.com/veg/hyphy-analyses/tree/master/BUSTED-MH) which implements an uncorrelated model of SRV across sites and includes multiple hit support. Applying this model to the alignments in the paper we find that the results are largely stable, and there is relatively little support for models with multi-nucleotide changes. We have added some discussion to the text and the supplementary information to clarify this point.

9. From figure 2 there appears to be evidence for selection in the pangolin CoV cluster within Orf1ab. While not the focus of this paper does this not suggest that there may be other adaptations under selection and responsible for its emergence in this animal host?

This is an interesting point that we had not originally mentioned in the text because of the little information we have about the pangolin CoVs and the few conclusions this observation could lead to. We now include this observation in our discussion, in relation to the reviewer's above comments regarding adaptation in minks. We point out that while branches leading to the pangolin CoVs show some evidence of adaptation, alluding to a story similar to what has been observed in minks, that is not the case for the terminal branch leading to SARS-CoV-2.

10. The title while scientifically accurate is a bit clumsy to me. I would suggest something catchier for a reader.

We agree with the reviewer. We have now changed the title to the version suggested by Reviewer 2.

11. How did the authors consider genomes with Ns in collapsing sequences into unique haplotypes. Were they ignored and only A,C,G,T characters considered?

Four-fold ambiguous characters (N), and only those ambiguous characters (i.e., not R,Y etc) were treated permissively, as matching any resolved character, during "unique haplotype" collapse. This has also been added to the Methods text.

Reviewer #2:

This important paper explores the evolutionary selective pressure on the Sarbecovirus subgenus of viruses and a subset of early SARS-CoV-2 genomes circulating in the human population in order to assess whether natural selection facilitated SARS-CoV-2's cross-species transmission to and consequential spread in the human population. In particular, the authors undertake the following key analyses:

1. Exploration of natural selection in SARS-CoV-2 (hereafter, SC2 for simplicity) human genomes.
First, the authors analyze, ~50000 human genomes of SC2, up until June 28, 2020 of the pandemic, limiting their analysis to genomes >29000 bps, with <1% divergence from the reference, with <0.5% ambiguous bases, and lacking stop codons. They use this dataset to assess evidence of purifying and/or positive selection in SC2, finding that most genetic variants in the virus occur at low frequency (in <15 genomes) and exhibit weakly purifying selection, consistent with a model of exponential virus growth. A few exceptions occur in the case of a few high frequency variants found in >3000 genomes that show weakly positive selection.

The authors undertake a number of advanced analyses to validate these few SNPs that are deemed to be under positive selection, identifying 10 candidate mutations with dN/dS ratios >1 that could be positively selected. They consider each of these individually, investigating the timing and laboratory of submission, the possibility of sequencing error or recombination to give rise to this variant and ultimately converge on four mutations that appear to be truly under positive selection: RdRp 323, S943, S614, and S141.

2. Analysis of selection in SARSr-CoV sequences in bats.
For the second major analysis of the paper, the authors analyze a subset of 19 non-recombinant regions of several bat/human/pangolin SARSr-CoVs identified in Boni et al 2019. Each non-recombinant region is analyzed independently, and for each region, the authors separate the viruses into an nCoV clade, representing those most closely related to SC2 and forming a monophyly and a non-nCoV clade, representing those more distantly related.

Using the program aBSREL, the authors first search for positive selection on specific branches in the SARSr-CoV phylogeny and find its imprints in the deepest branches of the nCoV lineage long before the emergence of the virus to humans, suggesting that it was not recent selection that allowed for the cross species shift.

The authors next use a program called BUSTED and their own extension to explore synonymous rate variation (SRV) across SARSr-CoV genomes, finding positive selection in the nCoV clade in Orf1ab, Spike and N proteins. Using the MEME method, the authors identify 85 particular sites in the nCoV clade under selection, most in Orf1ab, Spike, and N. Critically, they show a higher than expected proportion of sites in Orf3a, for which the function is not known, but they suggest it might play a role in immune evasion.

The authors' extension to the BUSTED method allowed them to infer differing substitution rate classes across the non-recombinant sites in the genome; the authors determine up to 200-fold differences in the rate of synonymous substitution across sites in the genome,

suggesting that some synonymous sites may be under strong purifying selection to purge deleterious mutations.

3. CpG depletion in the nCoV clade
The third and final major analysis of the paper investigates the depletion of CpG sites in the nCoV clade. CpG depletion is believed to be advantageous for virus evolution because It aids in evasions of a CpG-targeted mammalian immune response involving Zinc-finger Antiviral Protein (ZAP), as well as antiviral C to U hypermutation carried out by APOBEC3 cytidine deaminases. The authors use a framework called Synonymous Dinucleotide Usage (SDUc) to compare CpG representation across the 19 non-recombinant regions of the SARSr-CoV clade, finding significant CpG under-representation in Orf1ab for all Sarbecoviruses and lower CpG content overall in the nCoV clade Sabecoviruses vs all others. They fit this trait on two alignments of the 19 non-recombinant regions of the SARSr-CoVs to identify points where the "CpG suppressive" trait evolved: in particular, on the lineage leading to the nCoV clade.

Finally, they tested a model which allowed for a relaxed mutation rate in different clades to find evidence of an elevated substitution rate on the nCoV lineage subsequent to this CpG depletion event, giving way to a generalist virus clade.

General comments:
Though familiar with the SC2 evolutionary history literature, I am not a phylogeneticist by training and cannot comment critically on the methods selected for recombination and selection analysis (aBSREAL, MEME, BUSTED, SDUc). These appear to be appropriate to me. More generally, however, I believe that this paper is an important and relevant contribution to the SC2 evolution literature and should be published soon in PLoS Biology. It is not, however, the most clearly written paper I have encountered, and I have a few suggestions for how the authors could make their findings more accessible.

We thank the reviewer for their thorough assessment of our manuscript and their positive and constructive feedback. We believe we have addressed all of the reviewer’s comments and have responded to each point below.

Title: Title is a bit of a mouthful. What about dropping the "not humans" bit and including the word "generalist" -- something to the effect of: "Natural selection of SARS-CoV-2 in bats created a generalist virus and highly capable human pathogen"

We thank the reviewer for this very useful suggestion. We have now changed the paper’s title to this version.

Intro: It would be helpful if you set up these three major analyses summarized above at the end of the intro (~Line 99). Because PLoS Bio requires results to come before Methods, it can often lead to slightly disjointed papers, but I think one quick sentence preparing the reader for the three major areas of focus to follow would help a lot. In addition, I would recommend being very explicit about the two datasets used in this paper and stating that analysis #1 refers to the 50000 human SC2 genomes while analyses #2 and 3 refer to the SARSr-CoV dataset (in fact, two versions of it as presented in Boni et al. 2020)

We appreciate the need for clarifying our methodology. We have now included a sentence at the end of the introduction that should hopefully clarify the different dataset to the reader before moving to the results section. Furthermore, as prompted by Reviewer 1, we have included a supplementary figure that should hopefully provide an accessible visual explanation of the methods we use for each dataset (S2 Fig).

Discussion: I think some discussion of the pros vs. cons of being a specialist vs. a generalist virus is warranted here. The paper suggests that the entire clade of Sarbecoviruses is a highly generalist clade which seems like it should be a majorly adaptive feature. Why then have they not outcompeted all of the other tradeoffs? A nod to the literature on specialism vs. generalism in host-pathogen coevolution would be appropriate.

We agree with the reviewer that the previous version of our manuscript did not include much discussion regarding the specialist/generalist debate, a rather complex area, especially when it comes to pathogens. We now include this point at the end of the second paragraph of our discussion and cite relevant literature. We could elaborate more on this topic if the reviewer thinks this would be helpful.

Figures

Fig 1: Fig 1 is largely appropriate for summarizing the analysis of human SC2 genomes and evidence of positive selection but Fig 1A could be improved: it shows 'Variant Frequency' on the x-axis, which I believe gives the number of genomes in the dataset for in which a given variant is found, by both the dN/dS ratio on the primary y-axis (dots) and the count of variants of this type in the dataset on the secondary y-axis. So I think it is saying that there are ~7000 individual variants which are unique to only one genome in the dataset and these have a mean corresponding dN/dS ratio around .85. Likewise, I see it as saying that there are <50 variants that are found in over 3000 genomes and that these have an average dN/dS ratio of around 1.4. Is this a correct interpretation?

We have now updated the analysis relating to Figure 1. We also made a couple of cosmetic changes to Figure 1, including changing the colours in Fig1A. Hopefully this version should be much easier to interpret.

Also, why is there no range of error for the bars (i.e. through bootstrapping) or the dots from the SLAC method?

We have added IQR bars to the SLAC method and revised all figure panels for clarity and to show analyses through Oct 12, 2020 and updated the results to include RdRp alongside S.

Fig 2. I found Fig 2 difficult to interpret. I have a few suggestions for how it could be improved:
1. Break it down into subsections (A, B, C) so that you can refer to each separately in the caption.
2. In the caption, explain the genome structure and positive/negative selection subset first, since these are at the top of the figure (and refer to this as figure component A).
3. The individual phylogenies are fine—just group these together as part B. I *think* the color scale for dN/dS ratio corresponds to the mean ratio inferred for the highlighted clades within the lineage, so if these are subgrouped together it will be easier to understand.

4. Then, discard the donut plots. It is unclear why they are only present for some of the ORF regions (presumably only those with significant positive selection are shown?) and also confusing that their color scheme is different from the phylogenies. Instead, just either refer to the supplementary table as is done anyway or make a small table as part C that lists omega3 parameter for each ORF from Table S4 and corresponding percentage and mention in the caption that omega1 and 2 were basically 0 for all parameters.

We have restructured the figure as suggested by the reviewer, and replaced the 'donut' figures with a supplementary Table of BUSTED parameters (S4 Table); this was also necessary to incorporate the changes introduced in response to Reviewer 1's comments regarding the impact of multiple nucleotide substitutions.

Fig 3. Lovely figure. Could be slightly easier to explain if you made the CpG values part B of the figure and the schematic part C (or if the schematic were a different figure entirely—they don't really relate to one another).

We thank the reviewer for the positive comment. We have now relabelled the figure as requested, panel B being the CpG presentation and panel C the schematic.

Fig 4. In general, very clear. Can you do some comparative stats on these SDUc values for the frame positions in these two regions of the spike protein (inset) to show that WuHan and RmYN02 don't differ in the nCoV half but do in the non-nCoV half?

Although the nature of the metric used here makes it a bit difficult to compare using classical statistical methods, which is why we avoided presenting such an analysis in the original manuscript, we have now performed an unpaired t-test on the absolute differences between SARS-CoV-2 and RmYN02 SDUc values, comparing the two regions of Spike and show a significant difference in CpG representation between them. The results are presented in the legend of the figure. The absolute differences between SDUc values of SARS-CoV-2 and RmYN02 for each frame position are significantly greater in the non-nCoV than in the nCoV region ($t_{2.07} = 3.03$, $p = 0.0450$; unpaired one-tailed t-test with unequal variance).

A few minor line-by-line comments here:

Line 43: change to "which created a relatively generalist"

This has now been changed as requested.

Line 47-50: wording is awkward. Change to "Evolutionary analysis identified this new virus to humans as a Severe acute respiratory syndrome-related coronavirus [1], in the Sarbecovirus subgenus of the Betacoronavirus genus, sister to the original SARS virus; it was subsequently named SARS-CoV-2 to reflect this relationship [2]."

This has now been changed as requested.

Line 62-63: it is not proven that SARS-CoV transmitted to humans via civets and the cited ref is just a review paper. Suggestion to change to "Later it became clear that while these animals may have been conduits for spillover to humans, they were not true viral reservoirs"

This has now been changed as requested.

Line 88: suggestion to change "us" to "humans"

This has now been changed as requested.

Line 144: would be good to here cite Plante et al 2020, now published in Nature

This has now been changed as requested.

Line 242: should be one sentence

This has now been changed as requested.

Line 359: why was 1% chosen as a cutoff for too divergent (I agree, as 1% divergence would far outpace the known mutation rate for SARS-CoV-2 but I think you should provide a ref indicating the reasonable range of expected divergence, especially up to the point in time studies in your data subset)

Unfortunately, it is quite difficult to find a reference for this cut-off. This is a rather standard, if arbitrary, threshold and we can assure the reviewer that it does not cause any trouble to our analysis. We hope that this explanation is sufficient.

Line 360: sudden shift to first person, present tense is perplexing. Please keep tense consistent throughout the paper. Past tense probably makes more sense (i.e. "the data from GISAID was filtered").

This has now been changed as requested.

Supplementary Materials:
Table S4 could be easily incorporated into the pdf file for the supplementary materials and would be more accessible that way.

We agree that this change will facilitate access to the table and have now moved it to the supplementary pdf.
